# Dose-Dependent and Non-Autonomous Signaling in CAKUT: A Lineage-Specific Framework from Conditional Knockout Studies

**DOI:** 10.3390/biom16030458

**Published:** 2026-03-18

**Authors:** Nela Kelam, Petar Todorović, Patricija Bajt, Nikola Pavlović, Tomislav Rakić, Katarina Vukojević, Anita Racetin

**Affiliations:** 1 Laboratory for Early Human Development, Department of Anatomy, Histology and Embryology, University of Split School of Medicine, Šoltanska 2A, 21000 Split, Croatia; nela.kelam@mefst.hr (N.K.); petar.todorovic@mefst.hr (P.T.); patricija.bajt@mefst.hr (P.B.); tomislav.rakic@mefst.hr (T.R.); katarina.vukojevic@mefst.hr (K.V.); 2 Center for Translational Research in Biomedicine, University of Split School of Medicine, Šoltanska 2A, 21000 Split, Croatia; 3 Department of Pathophysiology, University of Split School of Medicine, 21000 Split, Croatia; nikola.pavlovic@mefst.hr; 4 Laboratory for Cardiometabolic Research, University of Split School of Medicine, 21000 Split, Croatia; 5 Mediterranean Institute for Life Sciences (MedILS), University of Split, Meštrovićevo Šetalište 45, 21000 Split, Croatia

**Keywords:** CAKUT, conditional knockout, Cre-loxP, kidney development, nephron progenitor, mouse models, nephrogenesis

## Abstract

Background/Objectives: Congenital anomalies of the kidney and urinary tract (CAKUTs) represent the leading cause of pediatric chronic kidney disease, yet the molecular mechanisms underlying these malformations remain incompletely understood. While genetic studies have identified numerous CAKUT-associated genes, conventional knockout approaches often result in embryonic lethality or fail to reveal tissue-specific gene functions. This review aims to synthesize findings from conditional knockout mouse studies that have elucidated the spatiotemporal requirements of key signaling pathways during kidney development. Methods: We conducted a narrative synthesis of studies employing Cre-loxP conditional gene targeting in mouse models, identified through systematic searches of PubMed and cross-referencing of key primary research. Studies were selected based on their use of lineage-specific Cre drivers (*Six2*-Cre, *Hoxb7*-Cre, *Foxd1*-Cre) to investigate nephron progenitor maintenance, ureteric bud branching morphogenesis, and stromal–epithelial interactions. Results: Conditional knockout studies have redefined CAKUT pathogenesis as a disorder of dose-dependent signaling, temporal regulation, and inter-compartmental communication. WNT/β-catenin signaling operates in a biphasic, dose-dependent manner in nephron progenitors, with *Six2*-Cre-mediated β-catenin deletion causing premature progenitor depletion. *BMP* and *FGF* pathways demonstrate dose-dependent and context-specific functions in progenitor maintenance, while GDNF/RET signaling is essential for ureteric bud outgrowth and branching. Importantly, stromal-specific deletions have uncovered non-cell-autonomous mechanisms regulating nephron formation. Haploinsufficiency studies demonstrate that partial pathway disruption can reduce nephron endowment without overt CAKUT, predisposing to adult-onset hypertension and chronic kidney disease. Conclusions: Conditional gene targeting has mechanistically redefined CAKUT from a collection of structural malformations to a spectrum of disorders arising from quantitative perturbations in lineage-specific signaling networks. These findings establish that phenotypic severity is determined by the degree of pathway disruption, the developmental timing of insult, and the compartment affected, providing a framework for interpreting oligogenic interactions and variable penetrance in human CAKUTs.

## 1. Introduction

Proper formation of the urinary tract relies on complex, highly regulated, and controlled interactions among different embryonic tissues, including metanephric mesenchyme (MM) and the ureteric bud (UB). Mutations and environmental and other influences may disrupt well-organized developmental pathways, leading to congenital anomalies of the kidney and urinary tract, most commonly known as CAKUTs [1]. These anomalies refer to a wide range of structural malformations, including renal agenesis, renal hypoplasia, dysplasia, megaureter, horseshoe kidney, ectopic ureter, duplex collecting system, and related or similar phenotypes [2]. In addition to genetic modifications, epigenetic factors are increasingly recognized as vital contributors to CAKUTs. Consequently, CAKUTs show a variety of symptoms and genetic causes, ranging from single-gene defects to more complex interactions. This heterogeneity often makes it difficult to determine the exact molecular cause underlying these anomalies [3].

Alongside heart defects, congenital anomalies of the kidney and urinary tract, as reported by the EUROCAT database, represent one of the most frequent categories of malformations in humans, occurring in approximately 0.5–1% of newborns. Moreover, CAKUTs account for nearly 45% of pediatric and 7% of adult end-stage renal disease worldwide [4]. Clinically, CAKUTs may appear as an isolated condition or as part of a broader systemic disease with extrarenal involvement. These anomalies are generally discovered prepartum by ultrasonography, later in life following the development of various complications, or as an incidental finding [5]. Much of what is currently known about the biological mechanisms triggering CAKUTs, including the genes controlling renal development and nephrogenesis altogether, comes from research using genetically modified mouse models [6]. Such research models have enabled the identification of various genes that are potential causes of CAKUTs in humans. Notably, mutations in the *PAX2* and *HNF1B* genes in broad patient cohort studies have been recognized to play a key role in both isolated and syndromic forms of CAKUTs, accounting for roughly 15% of documented cases and consequently being of particular importance in diagnostic analysis [2]. Recent data suggest that CAKUTs are associated with a range of pathogenic genes, each of which correlates with a monogenic recessive or dominant form of the condition. Given the vast genetic locus heterogeneity and ongoing advances in sequencing methods, it is likely that many additional genes will be identified in the near future [6].

Standard knockout techniques, which involve deleting the genomic region encoding the target gene, are most commonly used to determine gene function in vivo. Knockout mouse models are created by replacing a functional gene with a nonoperating counterpart [7]. However, these conventional approaches present significant limitations for studying kidney development. In mice, approximately 25% of gene knockouts result in embryonic lethality [8], precluding analysis of gene function during later developmental stages, including nephrogenesis. Furthermore, global gene deletion cannot distinguish between cell-autonomous and non-cell-autonomous gene functions, nor can it reveal tissue-specific requirements in the complex multicellular environment of the developing kidney. Even when phenotypes in knockout mice are correctly interpreted as direct consequences of gene ablation, traditional knockout technologies have routinely overlooked important limitations, such as the presence of regions of genetic variability, including flanking and background genes [7]. These constraints necessitated the development of more sophisticated genetic tools capable of spatiotemporal control over gene inactivation.

The Cre-loxP system has emerged as a powerful solution to these limitations, enabling conditional gene targeting with precise spatial and temporal control. This binary system utilizes Cre recombinase, which recognizes and excises DNA sequences flanked by loxP sites, combined with tissue-specific or inducible promoters to achieve targeted gene deletion in specific cell populations at defined developmental stages [9,10,11]. In kidney research, the availability of Cre driver lines targeting nephron progenitors (*Six2*-Cre), ureteric epithelium (*Hoxb7*-Cre, *Ksp*-Cre), and stromal cells (*Foxd1*-Cre) has revolutionized our ability to dissect the cellular and molecular mechanisms underlying kidney morphogenesis [12].

While existing reviews have cataloged CAKUT-associated genes and described developmental pathways in broad terms, few have systematically examined how conditional knockout approaches have reshaped the mechanistic understanding of these malformations through compartment-specific analysis. Despite the identification of over 50 CAKUT-associated genes, a fundamental gap persists between gene discovery and mechanistic understanding: conventional knockouts frequently cause embryonic lethality or produce compound phenotypes that obscure cell type-specific functions. Conditional gene targeting has begun to close this gap by enabling the dissection of gene function within individual renal lineages at defined developmental stages. This review comprehensively examines how conditional knockout studies have illuminated the roles of key signaling pathways—including WNT/β-catenin, BMP, FGF, and GDNF/RET—in nephron progenitor maintenance, ureteric bud branching, and stromal–epithelial crosstalk, establishing a lineage-centered framework for understanding how disruption of these pathways at specific developmental stages and in distinct cellular compartments contributes to the CAKUT spectrum. Such a synthesis is timely given emerging evidence that identical pathways perform fundamentally different functions across compartments, that stromal signals exert non-cell-autonomous control over nephrogenesis, and that critical questions remain regarding how oligogenic pathway interactions produce the variable penetrance characteristic of human CAKUTs. Although important differences exist between murine and human nephrogenesis, which are discussed in Section 4.7, mouse conditional models remain the most tractable system for dissecting lineage-specific gene function in kidney development.

## 2. Kidney Development and Cell Lineages

**Figure 1:**
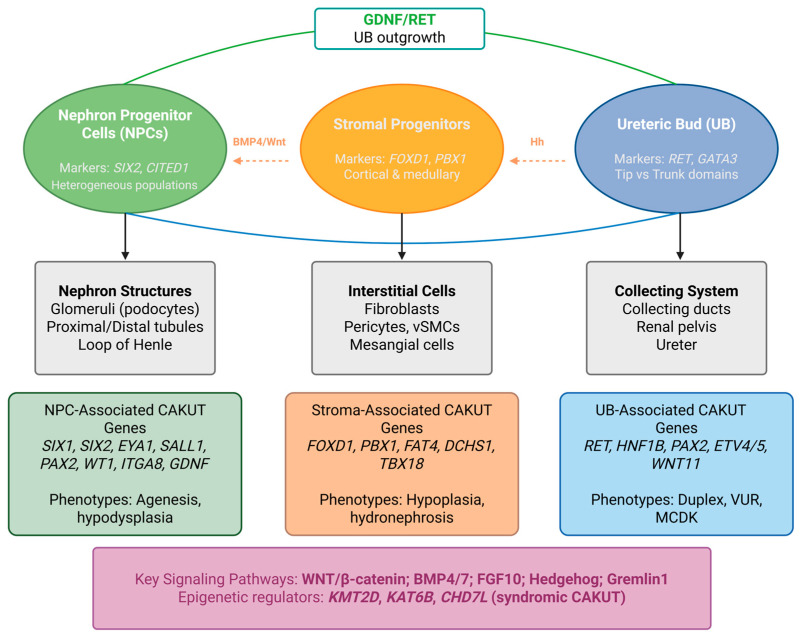
Progenitor populations, signaling interactions, and lineage-associated CAKUT genes in kidney development. The developing kidney consists of three principal progenitor compartments: nephron progenitor cells (NPCs), stromal progenitors, and the ureteric bud (UB). NPCs (a heterogeneous population) generate nephron epithelia, stromal progenitors form interstitial lineages, and the UB gives rise to the collecting system. Reciprocal signaling between compartments governs morphogenesis. GDNF, expressed in the metanephric mesenchyme/NPCs, activates RET in the UB to promote branching. Canonical Hedgehog signaling originates in the UB and acts on the stroma, which, in turn, secondarily regulates NPC maintenance via downstream mediators such as the BMP4 and Wnt pathways. Stromal-derived *Wnt5a* and *Cxcl12* further contribute to inter-compartmental communication. *PAX2* is expressed in both NPC and UB lineages. Lineage-associated gene defects result in distinct CAKUT phenotypes: NPC genes (*SIX1*, *SIX2*, *EYA1*, *SALL1*, *PAX2*, *WT1*, *ITGA8*, *GDNF*) are linked to renal agenesis and hypodysplasia; stromal genes (*FOXD1*, *PBX1*, *FAT4*, *DCHS1*, *TBX18*) to hypoplasia and hydronephrosis; and UB genes (*RET*, *HNF1B*, *PAX2*, *ETV4/5*, *WNT11*) to duplex kidney, vesicoureteral reflux, and multicystic dysplastic kidney. Syndromic CAKUT is associated with epigenetic regulators, including *KMT2D*, *KAT6B*, and *CHD7*.

The mammalian kidney develops through a complex process of reciprocal inductive interactions between distinct progenitor populations. Understanding these developmental mechanisms is fundamental to deciphering the pathogenesis of congenital anomalies of the kidney and urinary tract. The metanephric kidney arises from two embryonically distinct tissues: the metanephric mesenchyme and the ureteric bud, both derivatives of the intermediate mesoderm [13]. Recent advances in single-cell RNA sequencing have provided unprecedented resolution of cellular heterogeneity within the developing kidney, identifying distinct subpopulations and transcriptional landscapes that govern nephrogenesis [14,15] (Figure 1).

### 2.1. Nephron Progenitor Cells

Nephron progenitor cells (NPCs), residing in the cap mesenchyme surrounding UB tips, constitute a self-renewing, multipotent population that gives rise to all epithelial components of the nephron, including glomerular podocytes, proximal tubules, loops of Henle, and distal tubules [16]. The transcription factor SIX2 is the master regulator of NPC self-renewal and is essential for maintaining these cells in an undifferentiated state [16]. Single-cell transcriptomic analyses of human fetal kidney have identified four distinct NPC subpopulations (NPCa-d), characterized by differential expression of known CAKUT genes including *EYA1*, *SIX1*, *SIX2*, and *ITGA8*, suggesting spatial and functional heterogeneity within the nephrogenic niche [15,17]. The balance between NPC self-renewal and differentiation is tightly regulated by opposing signaling pathways: *WNT9b* and FGF signals from the UB promote both proliferation and, at higher concentrations, differentiation, while BMP7 signaling through JNK-dependent mechanisms supports NPC survival and proliferation [18,19].

### 2.2. Ureteric Bud and Collecting Duct Development

The ureteric bud emerges from the Wolffian (mesonephric) duct and undergoes iterative branching morphogenesis to form the collecting duct system, renal pelvis, and ureter [13]. GDNF/RET signaling represents the central regulatory axis controlling UB outgrowth and branching. Glial cell line-derived neurotrophic factor (GDNF), secreted by the MM, binds to its receptor tyrosine kinase *RET* and co-receptor *GFRα1* expressed in UB tip cells, activating downstream signaling cascades essential for branching morphogenesis [20]. The cell type-specific requirements and dosage sensitivity of this pathway, revealed through conditional targeting, are discussed in Section 4.2.

### 2.3. Stromal Progenitor Cells

Stromal progenitor cells (SPCs), marked by FOXD1 expression, occupy the peripheral regions of the metanephros and differentiate into interstitial fibroblasts, pericytes, and mesangial cells [21]. Beyond structural support, stromal cells play essential regulatory roles in nephron formation through paracrine signaling. Recent studies have identified Hedgehog signaling in *Foxd1+* stromal progenitors as a critical regulator of nephron number; constitutive activation of Hedgehog signaling in embryonic renal stroma inhibits nephron formation through *Cxcl12* and *Wnt5a*-dependent mechanisms [22]. Single-cell multiomics approaches have further revealed that mutations in chromatin regulators can perturb kidney developmental trajectories by rewiring the gene regulatory landscape of stromal cells, thereby affecting stroma–nephron interactions, including those mediated by *Pbx1*, *Meis1*, and *Wnt5a* [23].

### 2.4. Key Signaling Pathways in Nephrogenesis

Multiple signaling pathways coordinate to orchestrate kidney organogenesis. Canonical WNT signaling plays dual roles in NPC self-renewal and differentiation [18,24], requiring precise dose-dependent responses to β-catenin transcriptional complexes [25]. BMP signaling exhibits context-dependent effects, with BMP7 supporting NPC maintenance and BMP4 restricting ectopic UB formation, balanced by the antagonist Gremlin1 [19,26,27,28]. FGF signaling provides complementary support for UB branching and progenitor survival [29]. The cell type-specific requirements of these pathways, revealed through conditional knockout studies, are examined in Section 4. Importantly, these pathways do not operate independently (Table 1). WNT and FGF signals converge on nephron progenitor maintenance, with FGF providing survival cues that enable progenitors to interpret WNT-mediated fate decisions [30]. BMP signaling intersects with both the GDNF/RET and WNT pathways: BMP4 restricts GDNF-responsive domains, while BMP7 maintains progenitor competence to respond to WNT signals [19,26,27,28]. However, several aspects of cross-talk remain unresolved, including whether FGF and BMP pathways function primarily as permissive survival signals or actively instruct fate decisions, and the extent to which these pathway hierarchies are conserved in human nephrogenesis.

### 2.5. Major Genes Implicated in CAKUTs

Whole-exome sequencing studies have identified monogenic causes in approximately 10–20% of CAKUT patients, with over 50 established disease genes and numerous candidate genes emerging from recent studies [42,43] (Table 2).

*HNF1B* mutations are among the most common monogenic causes of CAKUTs, frequently identified in patients with renal cysts and hypodysplasia [45]. *RET* mutations are found in a substantial proportion of CAKUT patients, reflecting the central importance of GDNF/RET signaling in kidney development [42,43]. Trio-based exome sequencing has identified de novo variants in novel candidate genes, including *SOX13*, in approximately 20% of CAKUT families [49]. Notably, genome-wide CRISPR screens in cultured NPCs have confirmed the involvement of multiple known CAKUT genes, including epigenetic regulators *KMT2D* and *KAT6B*, suggesting that dysregulation of NPC fates represents a significant source of kidney malformation [47].

### 2.6. Cell Type Specificity in Developmental Pathways

The integration of cell type-specific expression data with genetic findings has become essential for understanding CAKUT pathogenesis. Comparative single-cell analyses of human and mouse kidney development have identified both conserved and divergent features, including differences in progenitor niche organization and cell type-defining marker expression [14]. The observation that identical genetic mutations can produce highly variable phenotypes underscores the importance of cell type-specific gene function and cell–cell communication networks [46]. Recent advances in kidney organoid technology now enable functional interrogation of these pathways in human cellular contexts, with organoid-derived NPCs closely resembling primary human NPCs and enabling genome-scale studies of kidney development and disease [47,50]. These approaches have revealed that genome-wide CRISPR screens of NPCs identified hits overlapping with approximately 7.6% of known CAKUT-associated genes, consistent with dysregulated progenitor cell fates as a major contributor to congenital kidney malformations [47].

## 3. Cre-Driven Lines and Experimental Design

Cre recombinases are essential tools for studying kidney development and CAKUTs, as they enable cell type-specific, time-controlled gene deletion in mouse models via the Cre–loxP system. This approach allows researchers to precisely determine gene functions during nephrogenesis and later stages of urinary tract development, which is crucial for understanding CAKUT pathogenesis [12,51,52].

There are several Cre driver mouse lines available that can selectively target different kidney cell types during development or in the adult kidney, making them especially useful for modeling specific CAKUT phenotypes [52,53] (Table 3). The *Six2*-Cre line targets the cap mesenchyme, which contains nephron progenitor cells that give rise to all nephron epithelial cell types throughout nephrogenesis. This line is particularly effective for investigating genes involved in nephron progenitor maintenance and nephron number [16,54]. The *Hoxb7*-Cre line is used to target the ureteric bud and its epithelial derivatives throughout renal development. This line is used for studying ureteric bud branching morphogenesis and collecting system development [55,56]. *Foxd1*-Cre targets renal stromal progenitors, which give rise to all kidney mural cells, glomerular mesangial cells, and multiple interstitial cell types, including pericytes. The *Foxd1*-Cre line is particularly useful for studying stromal contributions to kidney morphogenesis and vascular organization [57,58]. In addition, Cre drivers are targeting fully differentiated nephron segments, such as *NPHS2*-Cre, which targets podocytes, and *Aqp2*-Cre, which targets collecting ducts. Together, these Cre driver lines provide a powerful toolkit for dissecting lineage-specific gene functions during kidney development, which is essential for understanding the diverse phenotypes seen in CAKUTs [52].

Spatial specificity alone is insufficient for CAKUT research, as many renal genes have distinct functions at different developmental stages [97]. Therefore, temporal control of recombination is also critical. Temporal regulation of gene expression can be achieved using CreERT2, a widely used inducible Cre system [51]. CreERT2 provides precise temporal control of gene recombination in mouse models for CAKUT research, as Cre recombinase is activated only after tamoxifen administration. CreERT2 is a fusion protein composed of Cre recombinase and a mutated ligand-binding domain of the human estrogen receptor (ERT2), which retains Cre in the cytoplasm until tamoxifen binding induces nuclear translocation and loxP recombination [98]. This temporal control system allows researchers to investigate stage-specific roles of renal genes in CAKUT pathogenesis, because gene recombination can be induced at a defined developmental time point, separating early embryonic functions from later developmental or postnatal roles [98,99].

### Experimental Design Using Cre-Driven Lines

**Figure 2:**
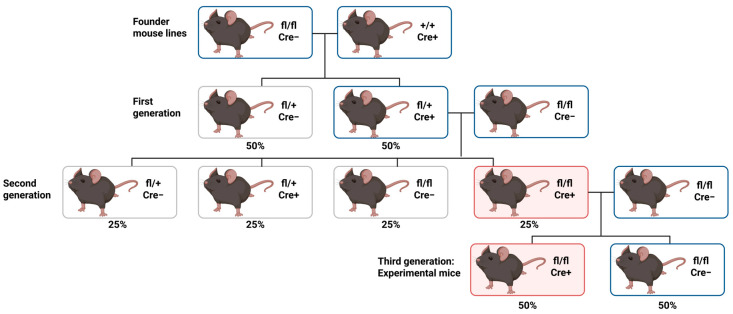
Founder mice consist of a homozygous floxed, Cre-negative line (fl/fl; Cre−) and a Cre-expressing line with wild-type floxed alleles (+/+; Cre+). These founders are then crossed to produce the F1 generation, which include 50% heterozygous floxed offspring with Cre recombinase (fl/+; Cre+) and 50% heterozygous floxed offspring without Cre recombinase (fl/+; Cre−). F1 animals that are fl/+; Cre+ are crossed with fl/fl; Cre− mice to generate the F2 generation. These crosses produce four genotypes in roughly equal proportions (25% each): fl/+; Cre-, fl/+; Cre+, fl/fl; Cre−, and fl/fl; Cre+. The fl/fl; Cre+ mice (highlighted in red in the diagram) are chosen for experimental use. These experimental mice are then crossed with fl/fl; Cre− animals to ensure homozygosity for the floxed allele in the next generation. The F3 generation consists of approximately 50% fl/fl; Cre+ mice, which are used as the experimental group, and 50% fl/fl; Cre− mice, which serve as controls.

Robust experimental design begins with thoughtful breeding schemes. Typically, homozygous floxed mice are crossed with Cre-heterozygous mice over successive generations to produce experimental animals that are homozygous floxed and Cre-positive, alongside Cre-negative littermate controls (Figure 2). Floxed alleles are maintained in homozygous form. At the same time, Cre lines are kept heterozygous to avoid potential haploinsufficiency or ectopic recombination [100,101]. Essential control groups include floxed mice lacking Cre, to control for effects of the targeting construct itself, and Cre-positive mice without a floxed allele, to control for Cre expression or toxicity [101].

Validation of recombination is a critical step. Reporter mice help detect Cre activity in vivo. They typically contain a loxP-flanked stop cassette upstream of a reporter gene such as lacZ or a fluorescent protein inserted at a locus such as ROSA26. While these reporter lines can reveal the Cre activity, they are not sufficient for full validation. Recombination must be confirmed directly at the target locus using PCR for deleted alleles, or via protein-level measurements such as flow cytometry or immunoblotting in purified target cells [102].

Several methodological limitations must be considered when interpreting conditional knockout studies. Recombination efficiency varies across genomic loci, meaning that reporter expression does not always indicate successful deletion of the target gene; validation must be confirmed directly at the target locus using PCR or protein-level measurements in purified target cells [102]. Incomplete recombination can produce a mosaic deletion, in which only a fraction of target cells undergo gene excision, potentially attenuating phenotypic severity and leading to an underestimation of gene function. Off-target somatic or germline recombination represents another concern, as Cre activity outside the intended cell population can complicate phenotype interpretation [101,103]. Cre toxicity itself, including DNA damage from excessive recombinase activity or disruption of endogenous gene regulation at the Cre insertion site, may alter cell biology independently of target gene deletion [104]. In inducible CreERT2 systems, temporal variability arises from differences in tamoxifen bioavailability, metabolism, and nuclear translocation kinetics, leading to variable recombination windows across individual animals and experiments [98]. These limitations can be mitigated by using single-copy Cre alleles, including appropriate control groups, validating recombination at the target locus, and employing complementary approaches such as multiple independent Cre lines targeting the same compartment [102].

Having established the methodological framework of conditional gene targeting, we now turn to examine how these approaches have elucidated the roles of specific signaling pathways in kidney development. We begin with the WNT/β-catenin pathway, which plays a central role in nephron progenitor cell fate decisions and has been extensively studied using conditional knockout models.

## 4. Key Findings from Conditional Knockout Studies

### 4.1. Nephron Progenitor Compartment: Six2-Cre Models

The nephron progenitor population, characterized by *Six2* expression, constitutes a self-renewing stem cell niche giving rise to all epithelial nephron segments [16,105]. Conditional knockout studies directed at this compartment have been particularly informative, as numerous genes critical for nephron progenitor maintenance cause early embryonic lethality when globally deleted, thereby precluding analysis of their renal-specific functions.

#### 4.1.1. Wnt/β-Catenin Signaling: Dose-Dependent Regulation

Canonical Wnt signaling, mediated through *β-catenin* (*Ctnnb1*), demonstrates how conditional approaches can reveal functions obscured by early lethality. Global *Ctnnb1* deletion in mice results in gastrulation-stage lethality [106,107]. *Six2*-Cre-mediated *Ctnnb1* deletion in nephron progenitors indicated that this molecule is required for progenitor maintenance; its absence causes premature differentiation, rapid depletion of the *Six2+* population, and severe renal hypoplasia [31].

Complementary gain-of-function studies, in which constitutively active β-catenin was expressed in nephron progenitors, produced the reciprocal phenotype: progenitors failed to differentiate, suggesting that β-catenin downregulation is required for differentiation [31,108].

Karner and colleagues provided evidence that the Wnt/β-catenin response operates in a biphasic, dose-dependent manner modulated by *Six2* [18]. Low pathway activity, combined with *Six2*, promotes progenitor self-renewal, whereas elevated *Ctnnb1* activity, in the absence of *Six2*, induces differentiation. Consistent with these murine findings, rare *CTNNB1* variants have been identified in CAKUT patient cohorts, and exome sequencing studies have confirmed that variants in multiple WNT pathway components co-occur in affected families, supporting the dose-dependent model established in conditional models [42,43,49].

#### 4.1.2. Cell-Autonomous Requirements for Progenitor Maintenance

The homeobox transcription factor *Six2*, which defines nephron progenitors, is required cell-autonomously to maintain the undifferentiated state of the mesenchymal progenitor population. Global *Six2* deletion causes premature progenitor differentiation resembling the *Ctnnb1* deletion phenotype [105]. Intriguingly, *Six2* haploinsufficiency paradoxically increases nephron number by enhancing progenitor proliferation, revealing a dose-dependent separation between *SIX2*’s roles in nephron progenitor self-renewal versus proliferation [54]. This finding illustrates how conditional genetics combined with careful phenotypic analysis can reveal non-linear dose–response relationships relevant to human genetic variation. In human cohorts, *SIX2* variants have been identified in patients with renal hypodysplasia, and the dose-sensitive relationship between *SIX2* levels and nephron number observed in mice aligns with the variable expressivity reported in affected families [15,17,42,43].

Additional transcription factors essential for nephron progenitors have been identified through *Six2*-Cre-mediated deletion. *Wt1* is required for progenitor survival; global *Wt1* deletion results in bilateral renal agenesis characterized by apoptosis of the metanephric mesenchyme [109]. *Six2*-Cre-mediated *Wt1* deletion in nephron progenitors results in defective nephrogenesis with expansion of stromal progenitors, confirming cell-autonomous requirements [110]. Mechanistically, WT1 maintains FGF signaling while suppressing BMP/pSMAD activity, with loss of Wt1 producing a pro-apoptotic phenotype that can be rescued by exogenous FGF [111]. *Sall1*, a multi-zinc-finger transcription factor, provides another compelling example. *Six2*-Cre-mediated *Sall1* deletion results in severe progenitor depletion and apoptosis of differentiating nephrons [112]. Sall1 has dual functions: activating progenitor genes and repressing differentiation programs [113]. Notably, mutations in human *SALL1* cause Townes–Brocks syndrome, an autosomal dominant disorder characterized by renal hypoplasia and agenesis, underscoring the clinical relevance of these developmental mechanisms [114].

#### 4.1.3. Signaling Pathway Integration

Nephron progenitors integrate multiple microenvironmental signals, including Wnt, FGF, BMP, and Notch pathways. FGF signaling from the ureteric bud, primarily via FGF8 and FGF20, promotes progenitor survival and proliferation [30,36,115]. Conditional deletion of *FGF receptors* in nephron progenitors leads to increased apoptosis and depletion of the progenitor pool [116]. BMP signaling exhibits bimodal functions: BMP7 promotes progenitor proliferation and survival through MAPK signaling [19], while BMP-SMAD signaling primes progenitors for Wnt/β-catenin-induced differentiation by enabling their transition from the CITED1+ to the SIX2-only compartment [35].

Pathway integration was elegantly shown through genetic epistasis analysis combining conditional alleles of *Ctnnb1*, *Pten*, and BMP/Notch signaling components [117]. These investigations revealed pathway convergence at multiple regulatory nodes, with context-dependent activation determining whether progenitors self-renew, differentiate, or undergo apoptosis.

Collectively, conditional studies targeting the nephron progenitor compartment reveal several generalizable principles. First, signaling pathways operate in a dose-dependent manner, with graded activity levels producing qualitatively distinct cellular outcomes. Second, developmental thresholds exist below which progenitor maintenance fails, yet above which differentiation is blocked, establishing narrow permissive windows for normal nephrogenesis. Third, individual transcription factors such as *Six2*, *Wt1*, and *Sall1* exhibit lineage-specific vulnerability, where even haploinsufficiency can alter nephron endowment without overt structural malformation. These principles provide a framework for interpreting the findings of the ureteric bud and stromal compartments described below.

### 4.2. Ureteric Bud and Collecting Duct: Hoxb7-Cre Models

The ureteric bud undergoes iterative branching morphogenesis to form the collecting duct system. *Hoxb7*-Cre and related driver lines have enabled systematic dissection of genes required for ureteric bud outgrowth, branching, and collecting duct differentiation.

#### 4.2.1. Compartment-Specific Functions of Wnt/β-Catenin Signaling

Analysis of β-catenin function in the ureteric lineage provides a striking example of context-dependent gene function. While β-catenin regulates self-renewal versus differentiation decisions in nephron progenitors, in the ureteric bud, its function is essential for branching morphogenesis. *Hoxb7*-Cre-mediated *Ctnnb1* deletion results in severe branching defects, yielding a simplified collecting duct system and renal aplasia or hypoplasia [32,33].

The mechanism includes both autonomous and non-autonomous effects. Cell-autonomously, β-catenin maintains ureteric bud cells in an undifferentiated precursor state, preventing premature expression of collecting duct differentiation markers and sustaining expression of branching morphogenesis genes, including *Ret* and *Wnt11* [33]. Non-autonomously, the ureteric bud signals to nephron progenitors via *Wnt9b*, which activates β-catenin-dependent differentiation pathways in the mesenchyme [118]. This dual-purpose function illustrates how a single molecule can coordinate development across compartments: the ureteric bud utilizes β-catenin to drive its own branching while simultaneously signaling to nephron progenitors, ensuring spatiotemporal coordination of nephron formation with collecting duct arborization.

Beyond regulating nephron progenitor cells in the metanephric mesenchyme, proper kidney formation requires coordinated development of the collecting system through ureteric bud outgrowth and iterative branching morphogenesis. These processes are critically dependent on GDNF/RET signaling, a pathway whose conditional dissection has revealed fundamental mechanisms of epithelial morphogenesis and provided direct insights into human CAKUT pathogenesis.

#### 4.2.2. GDNF/Ret Signaling and Branching Morphogenesis

The GDNF/RET pathway is essential for ureteric bud outgrowth and branching, and *RET*, *GDNF*, and *GFRA1* mutations account for a significant proportion of human CAKUT cases [2,42,119,120]. Global deletion of any pathway component results in bilateral renal agenesis due to failure of ureteric bud outgrowth [37]. However, dosage sensitivity and stage-specific requirements have been best revealed through conditional approaches.

Global *Ret* deletion results in severe ureteric bud branching defects and renal agenesis or hypodysplasia due to impaired ureteric bud outgrowth and branching [37]. Genetic dosage experiments demonstrated that branching extent is exquisitely sensitive to *Ret* signaling levels, with *Ret* heterozygous mice showing variable penetrance of renal defects and hypomorphic alleles causing intermediate phenotypes between normal and complete agenesis [38]. This dosage sensitivity has important clinical implications, as hypomorphic *RET* alleles may cause hypoplasia or vesicoureteral reflux rather than complete agenesis [120,121]. These findings are consistent with a developmental threshold model in which ureteric bud morphogenesis requires a minimum level of RET signaling activity to proceed normally. Above this threshold, branching proceeds with graded efficiency proportional to signal intensity; below it, morphogenesis fails progressively, ranging from mild branching deficits to complete agenesis. This threshold framework helps explain the phenotypic heterogeneity characteristic of human CAKUT, in which patients carrying different RET variant combinations, ranging from severe loss-of-function to subtle hypomorphic alleles, present with a corresponding spectrum of malformations rather than a single discrete phenotype. Importantly, this model aligns with the dose-dependent principles identified in the nephron progenitor compartment (Section 4.1), suggesting that threshold-sensitive signaling responses represent a shared vulnerability across renal developmental lineages.

#### 4.2.3. Transcriptional Regulators

Several transcription factors essential for ureteric bud development have been identified through conditional deletion. *Gata3*, which is mutated in humans and causes HDR syndrome (hypoparathyroidism, deafness, renal dysplasia), is required for ureteric morphogenesis and collecting duct differentiation [122]. *Hoxb7*-Cre-mediated *Gata3* deletion causes ectopic ureteric budding, hydronephrosis, and defects in nephric duct–cloaca fusion, demonstrating that these manifestations result from cell-autonomous nephric duct/ureteric lineage defects [123].

The studies described above have primarily focused on cell-autonomous gene functions in nephron progenitors or the ureteric epithelium. However, kidney development relies extensively on reciprocal signaling between epithelial and stromal compartments. Conditional gene targeting in stromal populations has uncovered essential non-cell-autonomous mechanisms that regulate both nephron formation and ureteric branching.

### 4.3. Stromal Compartment: Foxd1-Cre Models and Non-Autonomous Effects

The renal stroma was historically viewed as serving primarily structural and sustaining roles. Conditional knockout studies using *Foxd1*-Cre have fundamentally revised this view, revealing the stroma as an active signaling center that greatly influences epithelial development through paracrine mechanisms. These investigations have been fundamental to understanding non-autonomous gene functions and intercompartmental communication.

#### 4.3.1. Stromal β-Catenin: A Master Regulator of Inter-Compartmental Signaling

Stromal-specific *Ctnnb1* deletion demonstrates how conditional genetics reveal non-autonomous effects that established approaches would completely miss. *Foxd1*-Cre; *Ctnnb1^flox/flox^* mice show decreased nephrogenesis and abnormal nephron progenitor differentiation, despite nephron progenitors themselves retaining normal *β-catenin* expression [34]. The mechanism entails a multi-step signaling cascade: Stromal *β-catenin* maintains expression of factors that preserve *Wnt9b* expression in the adjacent ureteric bud; *Wnt9b* then signals to nephron progenitors to induce differentiation. Thus, *stromal β-catenin* loss leads to reduced ureteric bud-derived *Wnt9b*, which secondarily impairs nephron progenitor differentiation [34]. This three-compartment relay—stroma to ureteric bud to nephron progenitors—could only have been discovered through cell type-specific manipulation.

Complementary gain-of-function studies demonstrated that conditional stromal *β-catenin* activation causes ectopic accumulation of nephron progenitor-like cells and, in some contexts, lesions with features suggestive of nephroblastic proliferation [124]. These findings raise the possibility that dysregulated stromal signaling may contribute to aberrant progenitor expansion, although the relationship to nephroblastoma pathogenesis requires further investigation. These non-autonomous mechanisms have important clinical implications: patients carrying stromal gene variants may present with nephron deficiency or ureteric abnormalities that would not be predicted solely from the expression pattern of the mutated gene, complicating genotype–phenotype correlation in clinical CAKUT diagnosis.

#### 4.3.2. Hedgehog Signaling: Negative Regulation of Nephrogenesis

A paradigm-shifting discovery from conditional genetics is that stromal signals can negatively regulate nephrogenesis, limiting final nephron endowment [22,41]. *Foxd1*-Cre-mediated deletion of *Ptch1*, the Hedgehog pathway negative regulator, results in constitutive stromal Hedgehog activation. Counterintuitively, this led to reduced rather than increased nephron number [22].

The mechanism entails non-autonomous suppression of nephrogenesis through stromal-derived chemokines. Activated Hedgehog signaling upregulates *Cxcl12* and *Wnt5a*, which inhibit nephron progenitor differentiation [22]. This study established that the stroma actively modulates the extent of nephrogenesis, possibly acting as a regulatory mechanism to prevent premature exhaustion of the progenitor pool [22,41]. While Hedgehog pathway variants have not yet been systematically assessed in large CAKUT cohorts, the non-autonomous mechanism identified through conditional models predicts that stromal pathway variants could contribute to reduced nephron endowment without producing overt structural malformations detectable by current clinical screening.

#### 4.3.3. BMP Signaling and Stromal–Ureteric Bud Interactions

BMP signaling generally inhibits ureteric bud branching, while BMP antagonists promote it [26,27]. *Gremlin 1* (*Grem1*), a stromal-expressed BMP antagonist, is essential for normal branching morphogenesis [26]. Global *Grem1* deletion results in renal agenesis due to failure of ureteric bud outgrowth and branching; genetic reduction in Bmp4 levels restores branching morphogenesis, demonstrating that Gremlin1-mediated BMP antagonism is essential for normal branching [26,27,125].

The spatial distribution of stromal *Gremlin* expression—highest adjacent to ureteric bud tips—creates localized zones permissive for branching, while *BMP* expression in inter-branch regions potentially inhibits ectopic branching [26]. This spatial patterning by stromal signals helps explain the stereotyped geometry of the collecting duct tree. Beyond paracrine signaling, the stromal compartment also provides the extracellular matrix (ECM) microenvironment in which ureteric bud branching occurs. Emerging evidence suggests that matrix stiffness, composition, and remodeling dynamics can influence epithelial morphogenesis through mechanotransduction pathways. Consequently, conditional deletions that alter stromal cell identity or number may perturb not only paracrine signaling but also the mechanical properties of the peri-ureteric environment, potentially contributing to the branching geometry defects observed in stromal knockout models. Although mechanotransduction has not yet been directly examined through conditional approaches in the developing kidney, integrating biophysical frameworks with genetic models represents an important direction for understanding how stromal perturbations shape collecting duct architecture.

### 4.4. Cell Type-Specific Functions Revealed by Conditional Approaches

#### 4.4.1. Overcoming Embryonic Lethality

Many genes essential for renal development cause embryonic lethality when globally deleted [26,106,107,109]. *β-catenin* is the archetypal example: germline deletion is lethal at gastrulation [106,107], yet conditional deletion has demonstrated distinct, essential functions in nephron progenitors [31], the ureteric bud [32,33], and the stroma [34]. Without conditional genetics, the role of canonical Wnt signaling in kidney development would remain unknown.

#### 4.4.2. Context-Dependent Functions

Perhaps the most striking finding is that many genes perform entirely different functions depending on cellular context. The Wnt/β-catenin pathway demonstrates this principle across all three major renal compartments. In nephron progenitors, targeted using *Six2*-Cre, *β-catenin* maintains self-renewal at low activity levels while triggering differentiation at high levels, representing a biphasic, dose-dependent function [31]. In the ureteric bud, targeted using *Hoxb7*-Cre, *β-catenin* drives branching morphogenesis and regulates *Wnt9b* expression, serving a fundamentally morphogenetic function [32,33]. In the stroma, targeted using *Foxd1*-Cre, *β-catenin* regulates paracrine signals that maintain ureteric bud *Wnt9b* expression, representing a non-autonomous signaling function [34]. Thus, the same gene, acting through identical molecular pathways, performs three distinct biological functions depending on cellular expression context. This context dependency would be invisible to conventional knockout approaches.

#### 4.4.3. Temporal Requirements

Tamoxifen-inducible CreERT2 systems have revealed stage-specific requirements for developmental genes, demonstrating that the timing of gene inactivation profoundly influences phenotypic outcome. Lineage tracing using *Six2*-CreERT2 demonstrated that tamoxifen administration at E10.5 labels nephron progenitors that undergo self-renewal and contribute extensively to nephrons formed throughout development until E19.5, establishing that the *Six2+* progenitor pool is maintained by self-renewing cells rather than a separate progenitor population [16]. Similarly, *Cited1*-CreERT2 and *Eya1*-CreERT2 pulse-labeling experiments revealed that early-marked progenitors (E10.5–E12.5) undergo extensive self-renewal and contribute to nephrons over many days [66,126]. Temporal deletion studies using *Wnt4*-CreERT2 showed that cells expressing Wnt4 at E12.5 can revert to the progenitor state and contribute to nephrons formed 5–7 days later, while cells marked at E14.5 rarely return to the progenitor pool, indicating stage-dependent plasticity [127]. These temporal studies have established critical developmental windows and demonstrated that progenitor cells exhibit time-dependent changes in fate potential. Understanding these temporal requirements has important implications for interpreting human CAKUT phenotypes, as the timing of genetic or environmental insults during gestation determines both phenotypic severity and the specific kidney structures affected.

### 4.5. Non-Autonomous Effects and Cell–Cell Communication

Perhaps the most conceptually significant contribution of conditional knockout studies has been the revelation of the extent of non-autonomous gene functions and inter-compartmental signaling during kidney development.

#### 4.5.1. The Stroma–Ureteric Bud–Nephron Progenitor Axis

The three-compartment cascade linking the stroma, ureteric bud, and nephron progenitors represents the most striking example of non-autonomous signaling. Stromal β-catenin deletion causes nephron progenitor defects despite progenitors retaining normal *β-catenin* expression [34]. The discovery of these bidirectional signaling interactions has fundamentally changed our understanding of kidney development, shifting it from a linear inductive cascade to a complex reciprocal interaction network.

#### 4.5.2. The Nephron Progenitor Niche

The nephron progenitor niche, located at ureteric bud branch tips, is maintained by paracrine signals from both ureteric bud and stroma. Conditional genetics has systematically defined niche signal sources and targets [16,105]. *Wnt9b* secreted from the ureteric bud maintains progenitors in their undifferentiated state [18,24,118], while *FGF9*, derived from the ureteric bud, and *FGF20*, expressed within the mesenchyme, promote progenitor proliferation and survival [30,36,128]. The stroma contributes additional regulatory signals: Gremlin creates a BMP-inhibited zone permissive for progenitor maintenance [26,27,28], whereas retinoic acid, synthesized by stromal *Raldh2*, regulates ureteric branching through *Ret* expression [129,130]. These findings demonstrate that the nephron progenitor niche functions as a multi-signal integration center requiring simultaneous interpretation of Wnt, FGF, BMP, and retinoic acid, inputs from distinct cellular sources [39,40].

### 4.6. Refining Understanding of CAKUT Pathogenesis

Conditional mouse models have enabled the isolation of kidney-specific gene functions, clarifying the mechanistic bases of CAKUT phenotypes.

The integration of conditional knockout phenotypes with large-scale human sequencing data has increasingly validated the translational relevance of murine models. Whole-exome sequencing studies have identified pathogenic variants in over 50 CAKUT genes, with diagnostic yields of 10–27% [42,43,48,131], and many genes for which conditional models have demonstrated lineage-specific requirements, including *RET*, *PAX2*, *HNF1B*, and *SALL1*, are among the most frequently mutated in human patients [42,43,45]. The oligogenic interactions demonstrated in compound-heterozygous mouse models parallel observations that affected individuals frequently carry rare variants in multiple genes within the same developmental pathway [49]. Genome-wide CRISPR screens in cultured nephron progenitor cells have independently confirmed the involvement of multiple known CAKUT genes, providing convergent evidence from both conditional models and unbiased functional approaches [47]. However, variant burden analyses specifically stratified by lineage-specific functions, as established in conditional models, have not yet been performed, representing an important priority for future studies.

*RET* mutations account for a significant proportion of human CAKUT cases, ranging from bilateral agenesis to hypoplasia and vesicoureteral reflux [120,121,132]. Mouse conditional genetics demonstrated that graded reductions in *Ret* levels correspond to graded reductions in branching. Complete loss causes agenesis; partial loss causes hypoplasia; subtle reductions cause mild defects presenting as vesicoureteral reflux or low nephron endowment [133,134,135]. *PAX2* mutations cause renal coloboma syndrome [136,137]. While global *Pax2* deletion causes agenesis, conditional deletion in specific compartments revealed stage- and compartment-specific requirements [138,139,140]. *GATA3* mutations cause HDR syndrome [122]. Ureteric bud-specific *Gata3* deletion recapitulates renal phenotypes [123], confirming that kidney malformations result from ureteric bud-specific dysfunction rather than multisystem effects.

Collectively, these conditional findings support a reconceptualization of CAKUTs as a quantitative signaling disorder rather than a binary genetic defect. Rather than a simple presence-or-absence model, kidney malformations arise when aggregate signaling activity within or across developmental compartments falls below critical thresholds required for normal morphogenesis. This framework accounts for several hallmarks of human CAKUT that are difficult to explain through single-gene models: variable penetrance reflects individual variation in signaling pathway activity around threshold boundaries; variable expressivity arises because different structures have distinct threshold sensitivities; and oligogenic inheritance emerges when individually tolerable variants in multiple pathway components combine to reduce aggregate signaling below critical levels.

Many developmental genes in kidney formation show dosage sensitivity, with heterozygous loss causing milder phenotypes than homozygous loss, mirroring incomplete penetrance in human CAKUTs. For instance, *Gdnf* heterozygous mice (*Gdnf^+/−^*) exhibit low-penetrance renal defects (7–10% show renal agenesis or hypoplasia) compared to the complete bilateral renal agenesis in *Gdnf^−/−^* homozygotes [29,141]. Similarly, *c-ret* haploinsufficiency causes milder ureteric bud branching defects than complete loss, which results in severe renal agenesis or hypodysplasia [38]. Individuals carrying heterozygous loss-of-function mutations might exhibit subclinical phenotypes (mildly reduced nephron number) that manifest only under additional stress or in combination with variants in other genes.

Many CAKUT cases involve multiple genetic variants contributing modest effects. Conditional genetics enables testing of oligogenic models. Mice heterozygous for both *Fgf10* and *Gdnf* exhibit more severe phenotypes than single heterozygotes, with 81% showing absent or severely delayed ureteric buds at E11.5–E12.5 and 58% showing renal agenesis at birth, compared to 7–10% defects in *Gdnf^+/−^* single heterozygotes and normal kidneys in *Fgf10^+/−^* mice [29]. This demonstrates genetic interaction and supports the hypothesis that human CAKUT frequently results from the accumulation of rare variants affecting the same developmental pathway [142].

Conditional knockout studies have established that subtle developmental perturbations, insufficient to cause overt CAKUTs, can reduce final nephron number, predisposing individuals to hypertension and chronic kidney disease in adulthood. This evidence validates the Brenner hypothesis linking low nephron endowment to disease risk [143,144]. *Gdnf* heterozygous mice demonstrate this principle: they have reduced nephron numbers and develop elevated arterial pressure with age, despite normal kidney morphology at birth [145]. Stromal Hedgehog pathway activation also reduces nephron number through non-autonomous effects on nephron progenitor differentiation, demonstrating that stromal–epithelial signaling perturbations can affect final nephron endowment [22,41]. This evidence suggests that genetic variants modulating developmental signaling pathway activity—even without causing overt CAKUTs—could contribute to the wide variation in human nephron number and associated disease risk.

Collectively, these conditional knockout studies have not only identified essential genes and pathways for kidney development but have also revealed that subtle perturbations—insufficient to cause overt CAKUTs—can nonetheless impact nephron endowment and long-term renal health. These insights set the stage for considering how advances in genomic technologies, human pluripotent stem cell models, and therapeutic development may translate basic developmental biology findings into clinical applications.

### 4.7. Translational Limitations of Conditional Knockout Models

While conditional knockout studies have provided invaluable mechanistic insights, several limitations must be considered when extrapolating findings to human CAKUTs. First, there are important differences between murine and human nephrogenesis. In mice, nephrogenesis is completed shortly after birth, whereas in humans it ceases by approximately 36 weeks of gestation, with no postnatal nephron formation [13]. Human kidneys also contain substantially more nephrons than mouse kidneys, and the relative duration and complexity of the nephrogenic program differ considerably. These interspecies differences mean that the developmental windows identified through temporally controlled murine models may not directly correspond to equivalent human gestational stages.

Second, incomplete recombination remains a significant concern. As discussed in Section 3, Cre-mediated deletion is rarely complete in all target cells, and mosaic gene inactivation may attenuate phenotypic severity, potentially leading to an underestimation of a gene’s contribution to kidney development. Conversely, developmental compensation, in which alternative pathways or paralogous genes are upregulated following conditional deletion, may mask functions that are non-redundant in human contexts, where compensatory mechanisms may differ [46].

Third, promoter specificity and ectopic Cre expression can complicate the interpretation of phenotypes. Although Cre driver lines such as *Six2*-Cre, *Hoxb7*-Cre, and *Foxd1*-Cre are widely used, their expression domains may not precisely recapitulate endogenous gene expression patterns, and low-level activity outside the intended compartment has been documented [101,103,104]. This is particularly relevant when phenotypes involve interactions between compartments, as non-autonomous effects may be confounded by unrecognized ectopic recombination.

Finally, conditional models predominantly examine the consequences of complete gene loss within targeted cells, whereas human CAKUTs more commonly involve hypomorphic variants, regulatory mutations, or oligogenic interactions that produce partial reductions in pathway activity. The binary nature of Cre-mediated deletion, either with or without genes, may therefore fail to model the quantitative signaling perturbations most relevant to human disease. Recent advances in kidney organoid technology and comparative single-cell analyses of human and mouse development [14,47,50] are beginning to address these translational gaps, enabling functional validation of conditional knockout findings in human cellular contexts.

## 5. Future Directions and Translational Perspectives

The emerging integration of advanced genomic technologies, high-resolution cellular profiling, and organoid engineering may reshape our approach and understanding of CAKUT diagnosis, mechanistic understanding, and therapeutic development, although translation into routine clinical practice remains gradual and heterogeneous across phenotypes [146].

Whole-genome sequencing (WGS) overcomes exome limitations by detecting noncoding variants and structural rearrangements, improving diagnostic yield beyond the current 10–27% [48,131]. It is expected that WGS will enable detection of variations in noncoding regions of the genome that may represent novel causes of CAKUTs [147].

Despite significant advances in translational technologies that have deepened our understanding of CAKUT etiology, progress lags behind that of many other genetic kidney disorders. The establishment of large CAKUT cohorts will be crucial for achieving the resolution required to attribute causality to abnormal kidney and urinary tract development [148].

Polygenic risk scores integrating rare variants in GDNF/RET/WNT/BMP/FGF might enable granular risk stratification and prenatal assessment. Conditional mouse models link human genetics to CAKUTs. *TBX6* has been identified as a potential genetic driver in patients with *16p11.2* deletions, and mouse allelic series confirmed that insufficient *Tbx6* dosage determines CAKUT phenotypes in a dosage-dependent manner [149]. These findings link human variation to experimental outcomes, offering a framework for understanding variable expressivity and guiding translationally informed risk assessment. Single-cell and spatial transcriptomics reveal unexpected nephron progenitor plasticity and critical cell–cell interactions. Epigenomic profiling (ATAC-seq, bisulfite sequencing) identifies disease-associated chromatin states and methylation patterns, while proteomics delineates pathway-specific signaling cascades [148,150,151,152,153].

Human-induced pluripotent stem cell-derived kidney organoids have been reported to achieve up to 80–90% efficiency in generating nephron progenitor cells with multi-segmented nephron structures. CRISPR/Cas9-edited isogenic lines harboring patient mutations enable patient-specific disease modeling; organoid phenotypes successfully predict human disease outcomes. Organoid xenografts improve maturity and vascularization, enabling patient-tailored drug testing and safety assessment. On the other hand, the main limitation of kidney organoids derived from human pluripotent stem cells is the absence of essential supporting cells—including vascular, stromal, and immune cells—which limits their ability to fully recapitulate complex kidney diseases [151,152,153].

Base editing and prime editing correct point mutations without double-strand breaks, reducing off-target effects. Kidney-targeted delivery via engineered AAV serotypes (AAV-LK03 and AAV2/9) may enable efficient transduction of nephron segments. High-throughput organoid screening can identify compounds that modulate GDNF/RET, WNT/β-catenin, and BMP pathways, with the repurposing of FDA-approved drugs offering a potential route for rapid translational application [135,148,154,155,156,157].

Multi-biomarker panels integrating genetic, epigenetic, transcriptomic, and proteomic data represent areas that can be actively explored to enable CAKUT patient stratification by disease severity and progression risk. Deep learning models achieve >80% accuracy in prenatal CAKUT detection; AI-driven analysis identified novel CAKUT genes (*ARID3A*, *NR6A1*). Environmental exposures (maternal medications, infections, nutritional deficiencies) influence kidney development alongside genetics. Epigenetic biomarkers detectable in circulating cell-free DNA enable serial monitoring and early intervention [158,159,160,161,162,163,164]. However, these approaches currently lack the level of validation and replication required for routine clinical implementation.

Systems-level spatial transcriptomics across developing organs reveal shared developmental modules that govern morphogenesis. Endothelial cells and pericytes play a critical role in nephrogenesis through paracrine signaling, highlighting components that could be incorporated into future kidney organoids. Precision medicine requires robust genetic discovery from large sequencing cohorts, functional validation in organoids, biomarker development, identification of therapeutic targets, and clinical trials optimized for rare diseases [159,165,166,167,168,169].

Critical gaps remain: approximately 25% of nephrogenesis genes have established CAKUT associations; the mechanisms underlying variable penetrance remain poorly understood; organoid vascularization requires further optimization; and genomic research in underrepresented populations is essential and needs to be expanded. The convergence of CRISPR-based gene therapy, small-molecule pathway modulation, and tissue engineering creates an unprecedented opportunity to diagnose, treat, and potentially prevent congenital kidney malformations, with the realization of this vision dependent on coordinated advancement increasingly driven by international collaborative networks reshaping nephrology practice [170,171,172,173,174].

These emerging technologies have the potential to narrow the gap between genotype and clinical phenotype, particularly in patients with unresolved genetic causes, and to improve the interpretation of noncoding, structural, and multigenic variations. Integrating developmental biology, studies on human tissues, and genomics will be crucial for overcoming current limitations, informing the rational development of targeted therapies, and enabling a more personalized approach to the management of CAKUTs [175].

## 6. Conclusions

Conditional gene targeting has fundamentally transformed our understanding of CAKUTs, moving from a collection of discrete structural malformations to a spectrum of disorders arising from quantitative perturbations in lineage-specific signaling networks. Through the systematic application of Cre-loxP technology with lineage-restricted drivers—Six2-Cre for nephron progenitors, Hoxb7-Cre for the ureteric epithelium, and Foxd1-Cre for stromal progenitors—the studies reviewed here have established several unifying principles that redefine CAKUT pathogenesis.

First, key developmental signaling pathways, including WNT/β-catenin, BMP, FGF, and GDNF/RET, operate in a dose-dependent manner, in which graded reductions in pathway activity produce graded phenotypic outcomes ranging from subtle reductions in nephron endowment to complete renal agenesis. This dose-dependency provides a mechanistic basis for the variable penetrance and expressivity that characterize human CAKUT.

Second, identical genes perform fundamentally different functions across cellular compartments. β-catenin exemplifies this principle, serving biphasic self-renewal and differentiation roles in nephron progenitors, driving branching morphogenesis in the ureteric bud, and mediating paracrine regulation in the stroma. These context-dependent functions could not have been resolved through conventional knockout approaches and underscore the necessity of compartment-specific genetic analysis.

Third, the discovery of non-cell-autonomous signaling cascades, particularly the stroma-to-ureteric bud-to-nephron progenitor relay, has revealed that kidney development depends on continuous reciprocal communication among all three progenitor compartments. This inter-compartmental dependency implies that genetic variants affecting one lineage can have phenotypic consequences in another lineage, complicating genotype–phenotype correlations in clinical settings.

Fourth, haploinsufficiency and compound heterozygosity studies have demonstrated that subclinical reductions in pathway activity, insufficient to produce overt structural malformations, can nonetheless diminish nephron endowment and predispose to adult-onset hypertension and chronic kidney disease. These findings bridge developmental biology with the Brenner hypothesis and expand the clinical relevance of CAKUT-associated genes beyond the pediatric setting.

Despite these advances, important gaps remain. Interspecies differences in nephrogenic duration, nephron number, and compensatory capacity limit the translational fidelity of murine conditional models. The binary nature of Cre-mediated deletion imperfectly models the hypomorphic variants and oligogenic interactions most prevalent in human disease. Furthermore, variant burden analyses stratified by the lineage-specific functions established in conditional models have yet to be systematically performed in large patient cohorts.

Looking forward, the convergence of conditional knockout findings with single-cell multiomics, kidney organoid platforms, and large-scale human genomic studies offers an unprecedented opportunity to validate and extend the mechanistic frameworks established in mouse models. Integrating lineage-specific functional data from conditional studies with polygenic risk assessment and patient-derived organoid phenotyping may enable more precise genotype–phenotype predictions, improved risk stratification, and ultimately the development of targeted therapeutic interventions for congenital kidney malformations.

## Figures and Tables

**Table 1:** Comparison of signaling pathway functions across the three principal renal compartments. The same pathway performs distinct, context-dependent functions in different cellular compartments, as revealed by conditional knockout studies. References indicate key studies demonstrating compartment-specific roles.

Pathway	Nephron Progenitors	Ureteric Bud	Stroma
WNT/β-catenin	Biphasic: low = self-renewal; high = differentiation [18,31]	Branching morphogenesis; maintains *Ret/Wnt11* [32,33]	Paracrine: maintains UB *Wnt9b* expression [34]
*BMP*	*BMP7*: progenitor survival; *BMP*-*SMAD* primes differentiation [19,35]	*BMP4*: inhibits ectopic branching [26]	*Gremlin1* antagonism creates permissive zones [26,27]
*FGF*	*FGF8/20*: progenitor survival and proliferation [30,36]	*FGF10/7*: supports branching [29]	Not well characterized
*GDNF/RET*	*GDNF* source (ligand production) [20]	RET receptor: branching and outgrowth [29,37,38]	Indirect regulation via retinoic acid/*Ret* [39,40]
*Hedgehog*	Target (inhibited by stromal Hh) [22]	Indirect effects	Negative regulator via *Cxcl12/Wnt5a* [22,41]

**Table 2:** Major CAKUT Genes Organized by Affected Cell Lineage.

Cell Lineage	Progenitor Markers	Key CAKUT Genes	Associated Phenotypes	Key References
Nephron Progenitor Cells (NPCs)	*SIX2*, *CITED1*	*SIX1*, *SIX2*, *EYA1*, *SALL1*, *PAX2*, *WT1*, *ITGA8*, *GDNF*	Renal agenesis, renal hypodysplasia, oligomeganephronia	[13,15,16]
Ureteric Bud (UB)	*RET*, *GATA3*, *SOX9*, *KRT8*	*RET*, *HNF1B*, *PAX2*, *ETV4*, *ETV5*, *WNT11*	Duplex kidney, VUR, MCDK, hydronephrosis, UPJO	[20,43,44,45]
Stromal Progenitor Cells	*FOXD1*, *PBX1*, *MEIS1*	*FOXD1*, *PBX1*, *FAT4*, *DCHS1*, *TBX18*	Renal hypoplasia, hydronephrosis, aberrant vasculature	[21,22,23]
Signaling Pathway Regulators	Multiple lineages	*BMP4*, *BMP7*, *GREM1*, *FGF10*, *FGFR2*, *WNT4*, *CTNNB1*, *SPRY1*	Variable: agenesis to hypodysplasia depending on pathway balance	[18,24,26]
Epigenetic/Chromatin Regulators	Multiple lineages	*KMT2D*, *KAT6B*, *CHD1L*, *CREBBP*, *EP300*	Syndromic CAKUT (Kabuki, KBG syndrome), variable expressivity	[46,47]
Multi-Lineage Transcription Factors	Context-dependent	*HNF1B*, *PAX2*, *SOX17*, *SOX13*, *GATA3*, *LHX1*	Broad spectrum: cystic kidneys, hypodysplasia, VUR, MCDK	[42,48]

Abbreviations: CAKUTs, congenital anomalies of the kidney and urinary tract; MCDK, multicystic dysplastic kidney; NPC, nephron progenitor cell; UB, ureteric bud; UPJO, ureteropelvic junction obstruction; VUR, vesicoureteral reflux. Note: Gene names are italicized per standard nomenclature. Many genes affect multiple lineages; classification is based on expression and functional studies, reflecting the primary site of action.

**Table 3:** Conditional mouse models for CAKUT research.

Promoter	Renal Expression	Extrarenal Expression	Reference(s)
*11Hsd2* (*11β-hydroxysteroid dehydrogenase-2*)	Principal cells of the collecting duct, connecting tubules	Amygdala, cerebellum, colon, ovary, uterus, epididymis, salivary glands	[59]
*Aqp2* (*aquaporin-2*)	Principal cells of the collecting duct	Testis, vas deferens	[60]
*Atp6v1b1* (*V-ATPase-B1*)	Collecting ducts (intercalated cells), connecting tubule	Not well characterized	[61,62]
*Bmp7*	Cap mesenchyme	Bone, brain, heart	[63]
*Cdh16/Ksp-cadherin*	Renal tubules, collecting ducts, ureteric bud, Wolffian duct, mesonephros	Müllerian duct	[64,65]
*Cited1*	Cap mesenchyme	Not well characterized	[66]
*Emx1*	Renal tubules (proximal and distal tubules)	Cerebral cortex, thymus	[67]
*Foxd1/BF2*	Stromal cells	Brain, retina	[68]
*Ggt1 (gamma-glutamyl transferase 1)*	Cortical tubules	Not well characterized	[69]
*HoxB6*	Metanephric mesenchyme	Lateral mesoderm, limb buds	[70,71]
*HoxB7*	Ureteric bud, Wolffian duct, collecting ducts, distal ureter	Spinal cord, dorsal root ganglia	[72]
*Kap (kidney androgen-regulated protein)*	Proximal tubules	Brain	[73]
*Klf3*	Collecting ducts	Gonads	[74]
*Nphs1 (nephrin)*	Podocytes	Brain	[75,76]
*Nphs2 (podocin)*	Podocytes	Not well characterized	[77]
*Osr2*	Condensing metanephric mesenchyme; glomeruli	Palatal mesenchyme	[78]
*Pax2*	Pronephric duct, Wolffian duct, ureteric bud, cap mesenchyme	Inner ear, midbrain, cerebellum, olfactory bulb	[79]
*Pax3*	Metanephric mesenchyme	Neural tube, neural crest	[80,81]
*Pax8*	Renal tubules (proximal and distal tubules) and collecting ducts	Thyroid	[82]
*Pdgfrb (PDGFR-β)*	Mesangial cells, vascular smooth muscles	Pericytes, vascular smooth muscles	[83,84]
*Pepck*	Proximal tubules	Liver	[85]
*Rarb2*	Metanephric mesenchyme	Not well characterized	[86]
*Ren1 (Renin)*	Juxtaglomerular cells, afferent arterioles, mesangial cells	Adrenal gland, testis, sympathetic ganglia, etc.	[87]
*Ret*	Ureteric bud, collecting ducts	Dorsal root ganglion, neural crest	[88]
*Sall1*	Metanephric mesenchyme (tamoxifen-inducible system)	Limb buds, central nervous system, heart	[89]
*Slc5a2/SGLT2 (sodium-glucose transporter 2)*	Proximal tubules	Not well characterized	[90]
*Six2*	Cap mesenchyme	Not well characterized	[16]
*Sox18*	Cortical and medullary vasculature	Blood vessel and precursor of lymphatic endothelial cells	[91,92,93]
*Spink3*	Medullary tubules (distal or connecting tubules?)	Mesonephric tubules, pancreas, lung, liver, gastrointestinal tract	[80,81,94]
*T (brachyury)*	Whole kidney (both ureteric bud and metanephric mesenchyme)	Panmesodermal	[30]
*Tcf21 (Pod1)*	Metanephric mesenchyme, cap mesenchyme, podocytes, stromal cells	Epicardium, lung mesenchyme, gonad, spleen, adrenal gland	[53]
*Umod (uromodulin/Tamm-Horsfall protein)*	Thick ascending loops of Henle	Testis, brain	[95]
*Wnt4*	Renal vesicles, nascent nephrons (comma- and S-shaped bodies)	Not well characterized	[16,96]

## Data Availability

No new data were created or analyzed in this study.

## Abbreviations

The following abbreviations are used in this manuscript:

AAV	Adeno-associated virus
ATAC-seq	Assay for Transposase-Accessible Chromatin sequencing
BMP	Bone morphogenetic protein
CAKUT	Congenital anomalies of the kidney and urinary tract
CITED1	Cbp/p300-interacting transactivator 1
CKD	Chronic kidney disease
Cre	Causes recombination (recombinase)
CreERT2	Tamoxifen-inducible Cre recombinase
CRISPR	Clustered regularly interspaced short palindromic repeats
CTNNB1	Catenin beta 1 (β-catenin gene)
DNA	Deoxyribonucleic acid
ESRD	End-stage renal disease
ETS	E26 transformation-specific
ETV4/5	ETS variant transcription factors 4/5
EYA1	Eyes absent homolog 1
FDA	Food and Drug Administration
FGF	Fibroblast growth factor
FGFR	Fibroblast growth factor receptor
FOXD1	Forkhead box D1
GATA3	GATA binding protein 3
GDNF	Glial cell line-derived neurotrophic factor
GFRα1	GDNF family receptor alpha 1
HDR	Hypoparathyroidism, deafness, renal dysplasia (syndrome)
Hh	Hedgehog
HNF1B	Hepatocyte nuclear factor 1 beta
iPSC	Induced pluripotent stem cell
ITGA8	Integrin subunit alpha 8
JNK	c-Jun N-terminal kinase
loxP	Locus of X-over P1
MAPK	Mitogen-activated protein kinase
MCDK	Multicystic dysplastic kidney
MET	Mesenchymal-to-epithelial transition
MM	Metanephric mesenchyme
NPC	Nephron progenitor cell
PAX2	Paired box 2
PBX1	PBX homeobox 1
PTCH1	Patched 1
PTEN	Phosphatase and tensin homolog
RET	Rearranged during transfection (receptor tyrosine kinase)
RNA	Ribonucleic acid
SALL1	Spalt-like transcription factor 1
scRNA-seq	Single-cell RNA sequencing
SIX2	Sine oculis homeobox homolog 2
SMAD	Suppressor of mothers against decapentaplegic
SPC	Stromal progenitor cell
SPRY1	Sprouty RTK signaling antagonist 1
TBX18	T-box transcription factor 18
TGF-β	Transforming growth factor beta
UB	Ureteric bud
UPJO	Ureteropelvic junction obstruction
VUR	Vesicoureteral reflux
WGS	Whole-genome sequencing
WNT	Wingless-related integration site
WT1	Wilms tumor 1
